# Domestic Summary

**Published:** 1839-11-09

**Authors:** 


### DOMESTIC SUMMARY.

*University of Virginia.*—Dr. Howard, late Professor in the Medical Department of the University of Maryland, has been appointed Professor of Medicine in the University of Virginia, and he has accepted the appointment.

*Death of Dr. Antony.*—We learn with much regret the death of Dr. MILTON ANTONY, Editor of the Southern Medical and Surgical Journal, and Professor of Obstetrics in the Medical College of Georgia—a physician of considerable professional distinction, an able writer, and a worthy gentleman.

*Southern Medical and Surgical Journal.*—We are sorry to see that the death of Dr. Antony has caused the suspension of this journal. Should future arrangements be made for the continuance of the work, due notice will be given to its subscribers.

*Medical College of Georgia.*—The vacancy in the Faculty of this Institution, created by the death of Dr. Antony, will, we learn, be filled at as early a day as practicable, and no interruption will take place in the regular course of lectures.

